# ERP evidence of attentional selection outside of effective oculomotor range

**DOI:** 10.1007/s00221-025-07219-0

**Published:** 2025-12-23

**Authors:** Wiktor Więcławski, Jakub Paszulewicz

**Affiliations:** 1 Institute of Psychology, Jagiellonian University, Romana Ingardena 6, 30-060 Kraków, Poland; 2 Doctoral School of Social Sciences, Jagiellonian University, Krakow, Poland

**Keywords:** Spatial attention, Eye abduction, Premotor theory of attention, Effective oculomotor range, N2pc, Visual search

## Abstract

The close link between visual attention and the oculomotor system is well documented. Within the selection-for-action framework, two perspectives exist. According to Visual Attention Model (VAM) attention is seen as a prerequisite for successful movement execution, though it is considered a distinct cognitive and neural process. By contrast, the premotor theory of attention (PMTA) argues that the beneficial effects of attention are fully accounted for by the system’s preparation for saccadic eye movements. From this standpoint, a central prediction emerges: attentional advantages should be confined to regions within the oculomotor range, since saccadic planning is not feasible outside those limits. A common way to examine this prediction is to present cues and targets in a hemifield beyond the oculomotor range, typically achieved by occluding one eye while abducting the other. Using this method, Smith et al. showed that in a visual search task, exogenous orienting is reduced in the temporal hemifield when the eye is abducted. They concluded that exogenous attentional orienting is constrained by the range of potential saccadic movements. In our study, we sought to replicate Smith et al.’s findings while extending the paradigm with EEG recordings—an approach not yet applied in this context. PMTA predicts that, under eye abduction, stimuli appearing in the temporal hemifield would yield diminished N2pc amplitudes. An ANOVA revealed no reduction of N2pc amplitude in the temporal hemifield. Taken together, our results support the growing body of evidence suggesting that visual attention is not strictly bound to the oculomotor range.

**Supplementary Information:**

The online version contains supplementary material available at 10.1007/s00221-025-07219-0.

## Introduction

The premotor theory of attention (PMTA) originally posited that covert orienting requires activation of oculomotor programs (Rizzolatti et al. 1987; Craighero et al. 2004; Craighero and Rizzolatti 2005). This strong claim was later challenged by evidence showing that top-down attentional selection can extend into regions of the visual field that lie outside the oculomotor range (Smith et al. 2004, 2010, 2012; Casteau and Smith 2020). A more conservative formulation now proposes that saccade preparation is specifically required for bottom-up orienting. In this account, exogenous attention depends on eye-movement programs because these programs bias competition between perceptual representations in response to stimulus saliency, whereas endogenous orienting can bias competition directly through higher-order factors such as expectations about target location, without motor system involvement (Smith and Schenk 2012).

The eye-abduction paradigm has been widely employed to investigate the relationship between the oculomotor system and spatial attention (Craighero et al. 2004; Smith et al. 2010, 2012; Hanning et al. 2019; Hanning and Deubel 2020). In this design, the participant’s non-dominant eye is patched, and either the trunk is rotated or the display is shifted laterally so that maintaining fixation requires maximal abduction of the viewing eye. Under these conditions, the temporal hemifield falls outside the effective oculomotor range, rendering saccade preparation to that region impaired, whereas the nasal hemifield remains accessible for eye-movement planning. Several studies using this paradigm have reported reduced attentional effects in the temporal hemifield during abduction (Craighero et al. 2004; Smith et al. 2010), findings that were interpreted as supporting PMTA.

Recent findings using the eye-abduction paradigm indicate that bottom-up orienting of visual spatial attention is not confined to the oculomotor range, contrary to the predictions of PMTA (Carrasco and Hanning 2020; Li et al. 2021a). Specifically, well-controlled behavioral studies using the eye-abduction paradigm—with strict fixation monitoring and precise control of abduction angle—have demonstrated robust attentional benefits in reaction time and discrimination performance for stimuli presented in the hemifield that is largely inaccessible to eye movements (Hanning et al. 2019; Hanning and Deubel 2020). These results are consistent with the Visual Attention Model (VAM), which also conceptualizes attention within a selection-for-action framework but treats attentional selection and motor programming as distinct processes (Schneider 1995; Deubel 2014). According to VAM, attentional selection is a prerequisite for successful action, and attention is invariably engaged during movement preparation; however, the model does not require motor-system involvement in visual spatial attention when no goal-directed action is executed.

In cueing tasks, the most recent behavioural evidence suggests that visual spatial attention remains intact despite experimentally induced disruptions of eye-movement programming in healthy participants (Carrasco and Hanning 2020; Hanning and Deubel 2020). However, in clinical populations with gaze-control impairments of diverse aetiologies, the evidence is more mixed. One recent study reported preserved cueing effects in at least some patients with gaze paralysis (Masson et al. 2020), whereas several earlier studies documented abolished cueing effects in clinical groups (Craighero et al. 2001; Smith et al. 2004, 2021; Gabay et al. 2010).

Visual search tasks have yielded at least one influential study reporting slower bottom-up detection of targets in the hemifield that is inaccessible to eye movements (Smith et al. 2014). This distinction is theoretically important, as PMTA and VAM make conflicting predictions about covert visual search outside the oculomotor range. VAM remains agnostic, whereas PMTA posits that exogenous visual search should be less efficient when targets appear in locations beyond the reach of eye movements.

So far, studies using the eye-abduction design have relied exclusively on behavioral measures. While informative, these measures are indirect and temporally coarse. EEG provides well-established neural markers of attentional selection that allow one to track the time course of spatial selection with millisecond precision. One such marker is the N2pc, an ERP component characterized by a greater negative deflection at posterior electrodes contralateral to a visual target compared to ipsilateral sites (Eimer 1996). Although there is ongoing debate about the precise functional role of the N2pc in visual processing (Mazza et al. 2009a, b; Foster et al. 2018; Feldmann-Wüstefeld et al. 2021), it is widely regarded as a reliable index of spatial attentional allocation, particularly in light of its robust replicability and considerable effect size (Constant et al. 2025).

Beyond the eye-abduction approach, several EEG studies have tested predictions of the PMTA, generally reporting results consistent with the theory (Eimer et al. 2005; Mathews et al. 2006; Van Der Lubbe et al. 2012). These studies typically focused on lateralized ERP markers such as the N2pc or lateralized readiness potentials, providing neural evidence that attentional orienting is closely tied to movement preparation.

To address the gaps in the eye-abduction literature, we conducted a conceptual replication of Smith et al. (2014) feature visual search study, augmenting it with EEG recordings to examine whether the attentional selection marker N2pc is affected by the presumed impairment in eye-movement planning. According to the VAM, we would expect comparable N2pc amplitudes across all hemifields, including the temporal hemifield when the eye is rotated. In contrast, the PMTA predicts a substantially reduced—or even absent—N2pc amplitude for targets presented in the temporal hemifield under abduction. Such reductions in N2pc amplitude should be paralleled by slower reaction times.

## Methods

### Subjects

We recruited 37 participants (mean age = 26.43, 14 male, 4 left-handed, 13 left eye dominant). All provided informed consent and were compensated financially. 4 subjects did not finish the procedure and therefore are not included in the statistical analysis. Sample size was determined based on recent influential replication (Constant et al. 2025). In this multi-lab investigation, all labs acquired significant N2pc with even less than 30 subjects.

### Aparatus and experimental setup

**Fig. 1:**
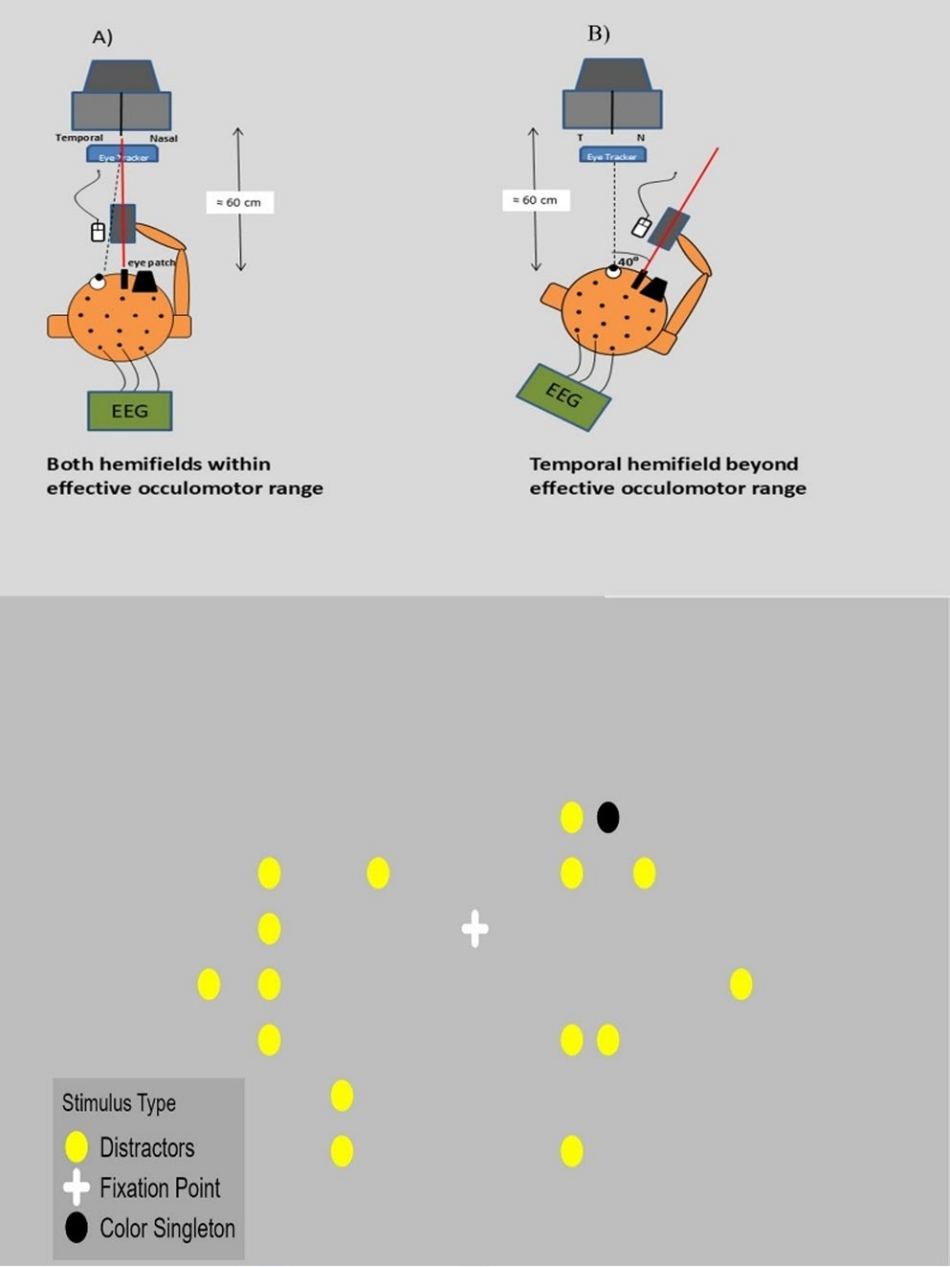
Schematic depiction of experimental design. *Note.* At the top panel eye frontal (**A**) and eye abduction (**B**) condition are presented. At the bottom panel an example experimental trial is shown

The experimental sessions took place in an acoustically shielded booth (Fig. 1). Subjects were seated approximately 60 cm away from the monitor. Subjects had their non dominant eye covered. Their heads were stabilized with a chinrest and the head position in the eye rotation condition was controlled by the experimenter with a video camera and corrected when needed. The stimuli were displayed on a 1920 × 1080 monitor on a grey background. The monitor had a refresh rate of 60 Hz. The gaze position was controlled by an Eyelink 1000 eye tracker.

#### Determining rotation angle

The rotation angle of the monitor was individually determined. We first rotated the ‘mobile board’ on which the monitor stood to a predefined position on the desk—corresponding to an angle of ≈40∘ (6,3 mean interpupillary distance divided by 2 was taken as reference to mark the position on the desk from which the angle was drawn. We then determined if the subject could be calibrated in that position. If successful, the board was moved further away and we tried calibration again. Maximal rotation angle in our set-up was approximately 45∘. We attempted to obtain maximal rotation angle for every subject.

### Visual search task

On the crossing of the vertical and horizontal mid-line of the monitor a white fixation cross was displayed which arms spanned horizontally and vertically ≈0.76∘ respectively. The targets and distractors were yellow and black circles with a visual angle diameter of ≈1.06∘. When the target in an experimental block was of yellow color the distractors where black and vice versa (in black target blocks). The target and the distractors appeared in random positions on two 5 × 7 grids that were located to the left and to the right of the fixation cross, displaced by ≈3.4∘ from it. The seven vertical positions of the grid started ≈3.4∘ above the horizontal midline and were separated from each other by ≈1.7∘. and extended to ≈6.8∘ below the horizontal midline. The five horizontal positions of the right and left grid started ≈3.4∘ off the vertical midline and were separated by ≈1.27∘, except the 5th most outer column that was ≈2.12∘ away from the fourth column. The color singleton targets could only appear at locations of the 3 inner columns of each grid.

There were 2 set sizes presented to the subjects: 8 and 16. Each set displayed was evenly divided between the right and left visual hemifield (between 2 grids). When the set size was 8 then 4 distractors appeared in random positions of one lateral grid and 3 distractors plus the singleton color target in random positions of the contrlateral grid. There were no target absent trials.

#### Experimental procedure

At the beginning of the experiment, the dominant eye of each participant was determined using the Miles Test (exactly like the one described in Więcławski et al. 2025). The subject’s non-dominant eye was patched. The within-subject design comprised two counterbalanced conditions: *Eye Frontal* (straight-ahead gaze) and *Eye Abduction* (head straight, dominant eye rotated to maintain fixation). The task and instructions were identical in both conditions. Participants were instructed to fixate centrally, monitor the display for a color singleton (750 ms), and respond within 1500 ms by pressing **B** (left) or **M** (right). They were asked to respond quickly and accurately; feedback was provided only when responses were too slow.

#### Block, trial and stimulus presentation

The experiment was controlled with *Experiment Builder*. Each eye-condition included four interleaved blocks of 90 trials (ABAB or BABA), with six practice trials per block. Across the experiment, participants completed 720 trials (672 experimental). Targets were black or yellow singletons among distractors of the opposite color; set size (8 or 16 stimuli) was randomized.

Eye-tracking calibration and validation were performed before the task, with drift correction as needed. Trials began with a fixation cross (blue, turning white after ≥ 500 ms fixation within a 1.75° boundary). Fixation duration varied randomly between 500 and 1500 ms (100 ms steps), followed by the search set (750 ms) and then fixation alone (750 ms). Participants had 1500 ms to respond. Trials were repeated after fixation loss or missed responses. The inter-trial interval was 2000 ms. Participants could pause the task at any time by breaking fixation.

### EEG recording

EEG signals were recorded using a Biosemi Active Two amplifier with 32 channels at a 256 Hz sampling rate, following the 10–20 system. Electrooculogram was recorded simultaneously from 4 electrodes placed on the temples and below and above not occluded eye.

### EEG preprocessing

Data preprocessing was performed in BrainVision Analyzer software. Mastoid channels were used as a reference. High-pass (0,05 Hz) and low pass (30 Hz) filters and Notch filter (50 Hz) were applied to all of the channels. We chose apriori electrodes PO3 and PO4 for analysis. We manually inspected those channels. No subject needed an interpolation. Independent Component Analysis (ICA) was applied. Independent components were inspected manually. Eye, heart and muscle-related components were identified by their time, frequency and topographical features.

Then the signal was segmented into 1 s epochs ranging from − 200 ms pre stimulus to 800 ms poststimulus, time-locked to the onset of the search display. Segments containing trials where subject’s eyes moved outside the permitted fixation area before their motor response were marked in the EEG recording and rejected from further analysis.

Subtractive baseline correction was applied using mean amplitude value form − 200 ms prestimulus period to 0 ms, i.e. stimulus onset). 200–300 ms time window was apriori chosen as a N2pc window. The average values for this window were used in statistical analysis. N2pc was computed separately for each hemifield by subtracting the ERP ipsilateral to the targets in the given hemifield from ERP contralateral to the these targets.

### Statistical analysis

To test our hypothesis, we conducted two repeated-measures ANOVAs with two within-subject factors, each with two levels: hemifield (nasal vs. temporal), experimental condition (frontal vs. abducted), and their interaction. We performed two planned comparisons. The first comparison of interest was between nasal and temporal hemifields in the rotation condition. The second concerned the temporal hemifield in the frontal versus rotated condition. The same model was applied to both reaction times and N2pc amplitudes. To verify that our task in fact elicited pop-out search we performed simple paired t test between two set sizes. To test whether accuracy differs between conditions we used mixed-effects logistic regression. All analyses were carried out in R using functions from the *rstatix* and *lme4* packages.

## Results

### Behavioral results

Subjects were accurate across all conditions: Frontal–Nasal (M = 86.8%, SE = 1.9), Frontal–Temporal (M = 84.1%, SE = 2.3), Abducted–Nasal (M = 81.7%, SE = 2.1), and Abducted–Temporal (M = 85.3%, SE = 2.3). The mixed-effects logistic regression revealed a significant main effect of hemifield, with slightly lower accuracy for temporal compared to nasal targets (OR = 0.76, *p* = 0.035). Eye abduction did not significantly reduce accuracy (*p* = 0.149), and the Hemifield × Rotation interaction was also nonsignificant (*p* = 0.350). Post hoc Tukey-corrected contrasts showed that the only comparison approaching significance was Nasal–Frontal vs. Temporal–Rotated (OR = 1.36, z = 2.30, *p* = 0.097).

Incorrect trials were discarded, and reaction times were trimmed within each participant (> 3.5 SD). Although the difference in mean RTs between set sizes 8 (M = 392 ms, SE = 5.09) and 16 (M = 390 ms, SE = 5.19) reached statistical significance (t(32) = 3.09, *p* = 0.004, d = 0.54), the effect size was negligible in absolute terms (≈ 2 ms). Thus, we conclude that the task successfully elicited pop-out search.

**Fig. 2:**
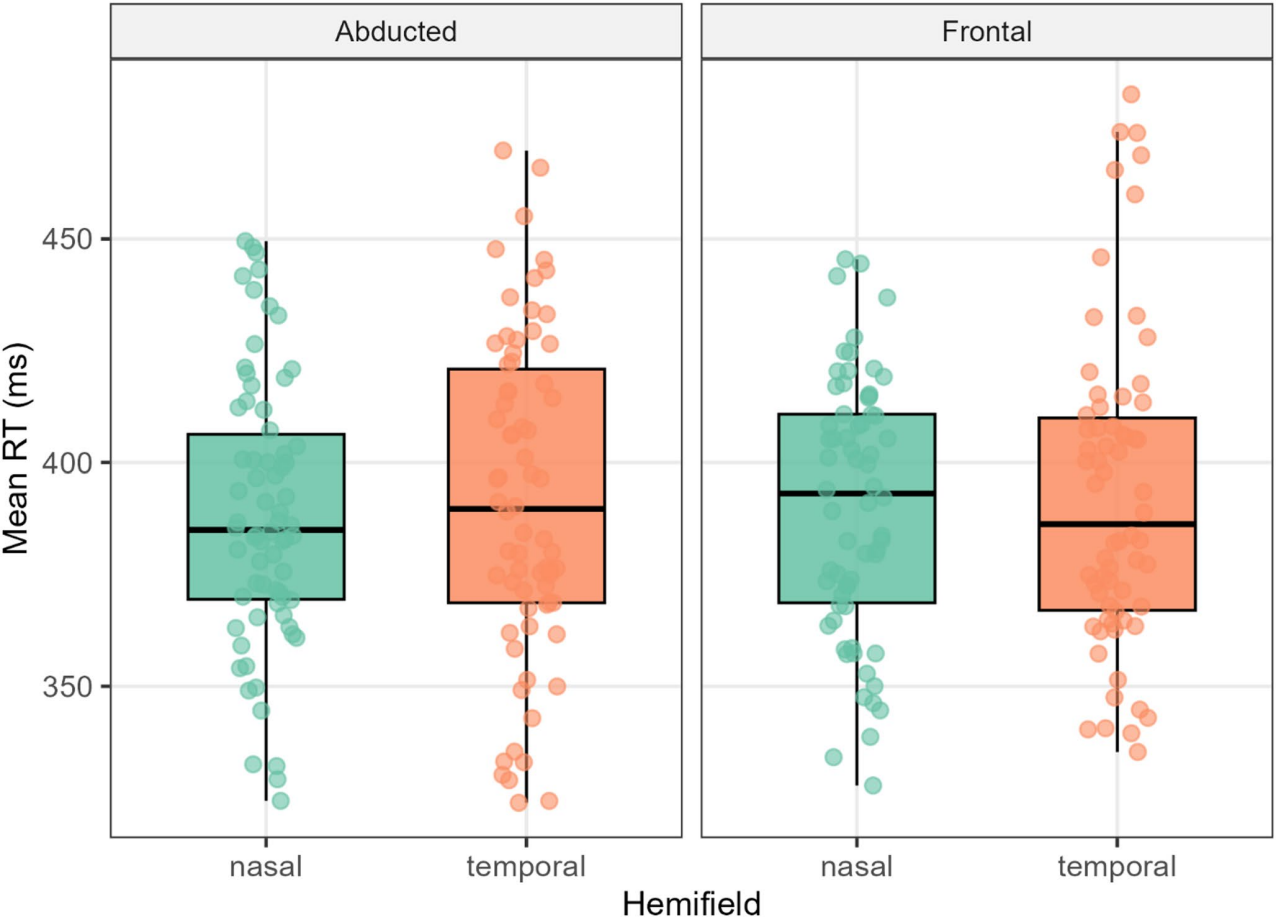
Reaction times for each experimental condition. *Note*. Reaction times are subject-level means from correct trials after per-participant trimming (> 3.5 SD). Boxes depict interquartile ranges with median lines; whiskers extend to 1.5 × IQR

ANOVA on reaction times revealed no evidence for differences between experimental conditions (Fig. 2). Neither the main effect of hemifield (F(1,32) = 0.50, *p* = 0.486, η^2^_p_ < 0.01) nor the main effect of experimental condition (F(1,32) = 0.05, *p* = 0.829, η^2^_p_ < 0.01) was significant, and the interaction was similarly absent (F(1,32) = 0.01, *p* = 0.906, η^2^_p_ < 0.01). Planned comparisons were likewise nonsignificant: Nasal–Abducted vs. Temporal–Abducted (t(32) = − 0.62, p = 0.543, d = − 0.07) and Temporal–Frontal vs. Temporal–Abducted (t(32) = 0.25, *p* = 0.808, d = 0.03).

### EEG results

**Fig. 3:**
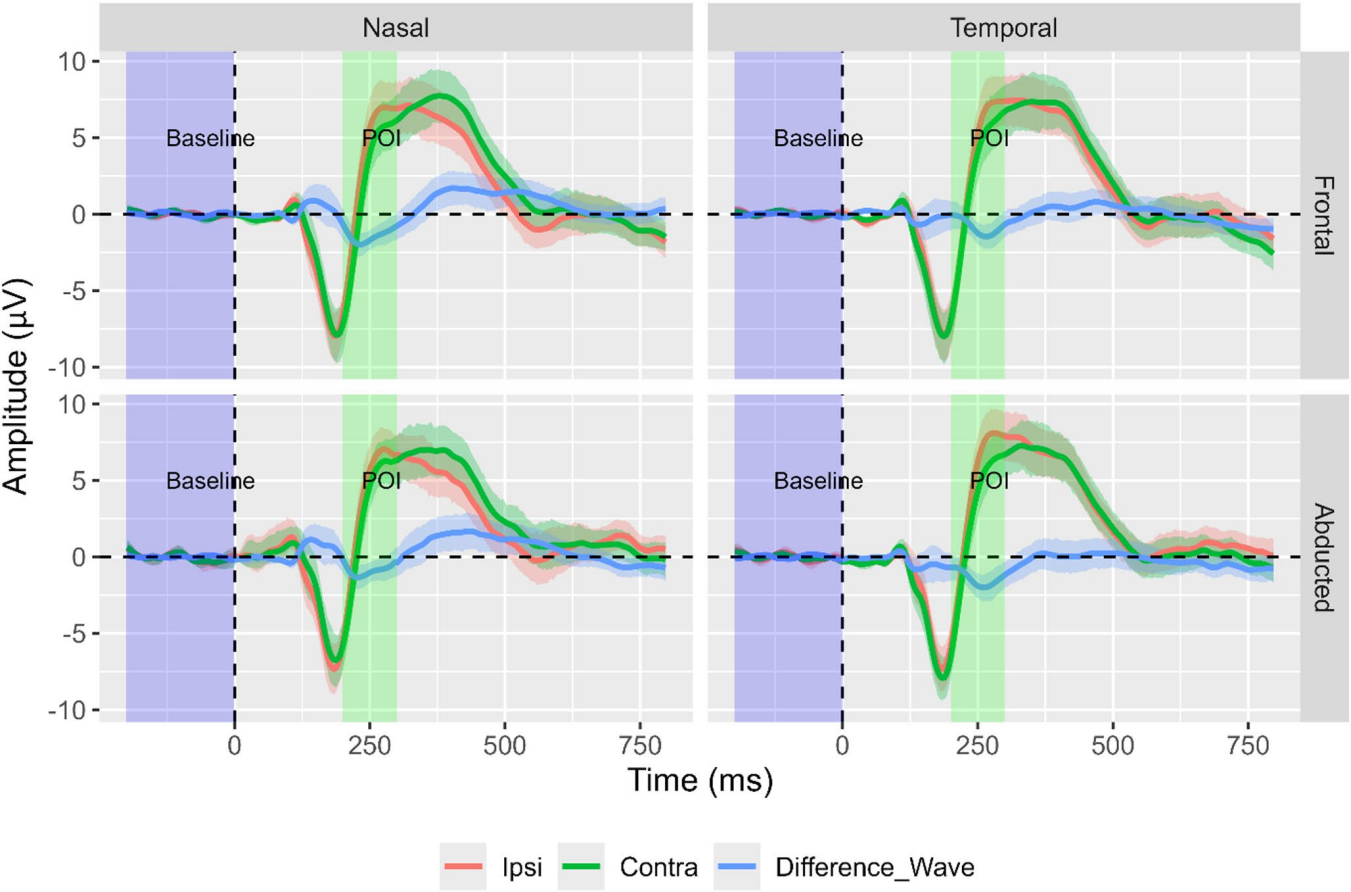
ERP plots in all experimental conditions with 95% bootstrapped confidence intervals. *Note.* Overall shape of ERP seems to be similar in all conditions at. Signal from channels PO3 and PO4 was used to create those plots

Visual inspection confirmed that we successfully elicited a typical N2pc component (Fig. 3). The ANOVA showed a significant interaction between hemifield and experimental condition (F(1,32) = 20.31, *p* < 0.001, η^2^_p_ = 0.39). Neither the main effect of hemifield (F(1,32) < 0.01, *p* = 0.994, η^2^_p_ < 0.01) nor the main effect of the experimental manipulation (F(1,34) = 0.07, *p* = 0.795, η^2^_p_ < 0.01) was significant.

Of the planned comparisons, one reached significance: N2pc amplitude in the temporal hemifield was larger in the eye-abduction condition compared to the frontal (non-rotated) condition (t(32) =  − 3.78, *p* < 0.001, d = 0.29). The second planned comparison did not reveal a significant difference in N2pc amplitude between nasal and temporal hemifields within the eye-abduction condition (t(32) = 0.90, *p* = 0.377, d = 0.28).

## Discussion

In this study, we attempted a conceptual replication of Smith et al. (2014). We conducted a simple singleton visual search experiment with EEG recording. We analyzed ERPs and found electrophysiological evidence suggesting that attentional selection outside the effective oculomotor range may be possible. N2pc amplitudes were similar in the nasal and temporal hemifields, regardless of eye position. The only significant difference in the planned comparisons was, in fact, completely opposite to the predictions of PMTA: the N2pc was even larger when the target was supposedly outside the effective oculomotor range. Reaction times were partially consistent with the electrophysiological results exhibiting no significant differences between conditions.

How should our results be interpreted, and why did PMTA fail to provide accurate predictions? Importantly, our findings should not necessarily be taken as evidence against PMTA. Some authors have argued that the preparation of movements with any effector can enhance the processing of relevant spatial locations (Van Der Lubbe and Abrahamse 2011; Perry et al. 2015; Perry and Fallah 2017). Current perspectives suggest that attention is not a single, unified mechanism but rather a collection of processes responsible for selecting and executing actions appropriate to the current environmental demands (Hommel et al. 2019). Various so-called priority maps have been identified within the parietal cortex (Zelinsky and Bisley 2015), each corresponding to specific actions or effectors (e.g., reaching, grasping, or eye movements). These maps provide spatial representations in formats most suitable for the respective effector.

For example, the Lateral Intraparietal Area (LIP) is organized retinotopically and appears particularly well adapted to guide the oculomotor system (Andersen et al. 1992; Bisley and Goldberg 2003). Priority maps encode the relative importance of stimuli in the environment, biasing competition among potential action programs and facilitating execution of the selected actions by enhancing processing of relevant sensory information (Bisley and Goldberg 2010; Klink et al. 2014; Zelinsky and Bisley 2015). Importantly, these maps integrate both bottom-up inputs, such as stimulus salience, and top-down signals, such as goals and expectations. A central top-down influence is motor preparation: activity related to upcoming movements shapes priority map activity and thereby modulates visual processing. However, psychophysical evidence indicates that this presaccadic attentional enhancement is not entirely reducible to covert attention, as the two can be dissociated on the basis of their properties (Li et al. 2021b).

Furthermore, both behavioural and neurophysiological studies provide numerous evidence for dissociation between oculomotor programming and attention (Gottlieb 2014). Behavioural data suggest that saccades and covert attention can be deployed to separate spatial locations (Wollenberg et al. 2018). Neurophysiological recordings show that LIP neuronal activity is more predictive of the location of a visual cue than of the location of an upcoming pro-saccade or anti-saccade (Gottlieb and Goldberg 1999), whereas key structures of the oculomotor system—the Frontal Eye Fields (FEF) and the Superior Colliculus (SC)—appear to exhibit the opposite pattern (Everling et al. 1999; Schall et al. 2011). From these empirical findings emerges the vision of the system where FEF and SC are mainly responsible for planning and execution of the eye movements. Those structures are biased by LIP to prefer the locations represented as important. Nevertheless, LIP does receive feedback from FEF as part of corollary discharge mechanism (Sommer and Wurtz 2008), and this feedback probably alter the priority. So, LIP maintains a dynamic priority map influenced by both external salience and internal motor plans, while FEF/SC implement the final motor decisions.

In line with priority map view of the role of parietal cortex in visual processing, there seems not to be any apparent reason why attentional selection should not be possible throughout the entire visual field, including regions beyond the effective oculomotor range, as long as these spatial regions are represented in at least one priority map within the parietal cortex. In the works that investigated the receptive fields of LIP neurons, there is no indication that oculomotor range plays any role in their activity (Gottlieb and Goldberg 1999; Gottlieb 2014). Importantly, in the case of eye abduction, there is no clear rationale to assume that the temporal hemifield of the abducted eye would not be represented within retinotopic priority maps. Furthermore, even if an eye-centered map was distorted in some manner, other effector-specific maps would likely remain unaffected. In other words, even if effective preparation of an eye movement is compromised, planning movements with alternative effectors (such as head or hand) may remain viable.

Indeed, it has been proposed that incorporating head rotations into the eye abduction paradigm could clarify this issue (Hanning et al. 2019; Hanning and Deubel 2020). We know that usually combined eye-head movements are planned to achieve peripheral targets (Guitton and Volle 1987; André-Deshays et al. 1988; Freedman et al. 1996). To date, eye-head abduction approach has been investigated only once, involving a single patient (Masson et al. 2020). In that study, a normal cueing effect was observed even within the hemifield that was ostensibly inaccessible to eye movements.

The most puzzling aspect of our results is the small increase in N2pc amplitude observed in the abducted temporal hemifield, which runs counter to our initial predictions. This finding is also inconsistent with a previous study that examined the neural basis of temporal hemifield advantages: using an eye-patching approach, Huber-Huber et al. (2015) reported a reduction in N2pc amplitude in the temporal hemifield. However, their results are not directly comparable to ours, as the task they employed differed substantially from the one used in the present study.

In our study, we treated the N2pc as an index of covert attentional selection. From this perspective, the key finding is the very presence of an N2pc in the abducted temporal hemifield, as it demonstrates that attentional selection can occur outside the effective oculomotor range. How can this unexpected increase be understood within our theoretical framework?

First, we must consider whether it represents a genuine effect. It is important to evaluate it in the context of the overall pattern of results. If this increase were cognitively meaningful, we would expect it to emerge consistently across relevant comparisons. Yet, the difference reaches significance only when comparing the abducted temporal condition to the frontal temporal condition, but not when comparing the abducted temporal to the abducted nasal condition. Had we observed significant differences in the same direction in both planned contrasts—particularly those that align with the behavioral effect—we would have stronger grounds to interpret this increase as theoretically meaningful and warranting further explanation.

Although some inconsistencies might be pointed out, we believe our results are in line with the rest of the most recent literature presenting good quality behavioral evidence on attentional selection outside of oculomotor range (Carrasco and Hanning 2020). Our research successfully attempted to close important gap in the literature on the link between oculomotor system and visual spatial attention.

### Limitations

Maintaining stable fixation at the limits of the oculomotor range is nearly impossible for many participants. In our experiment, subjects struggled to continuously fixate on the cross during the rotation condition and often looked straight ahead between trials to temporarily relieve muscle strain. One way to overcome this difficulty and ensure that some targets are truly outside the saccade range is to rotate the eye to a still comfortable position and then present temporal hemifield stimuli at a far peripheral location (Hanning and Deubel 2020), or to avoid head rotation altogether and place stimuli in the far periphery (Casteau and Smith 2020). Unfortunately, these approaches were not feasible for us, as N2pc amplitude decreases substantially when stimuli are presented more than 4° of visual angle into the periphery (Papaioannou and Luck 2020). Thus, although we attempted to restrict participants’ effective oculomotor range, we were not as successful in doing so as others.

Although we describe our study as a conceptual replication of Smith et al. (2014), our visual search task differed from theirs in several important respects. Smith et al. (2014) required participants to report the orientation of the target (a non-lateralized task), whereas in our version participants indicated the location of the target (a lateralized task). Both versions reliably elicit pop-out search; however, this procedural difference may have introduced unintended consequences visible in the ERP data (Supplement 1), which may or may not play a role in explaining the discrepancies between the two studies.

With regard to ERPs, tasks that require monitoring the spatial dimension of the response can elicit an additional lateralized component in a similar temporal window—namely, the N2cc (Cespón et al. 2012). Fortunately, this motor-related component does not appear to influence N2pc amplitude (Cespón et al. 2012). Supplement 1 includes topographical plots that suggest the presence of both N2pc and N2cc in our data. The absence of a clear posterior voltage peak typically observed in N2pc studies may reflect visuomotor integration processes (Panek et al. 2025), as our task involved predefined motor responses for each target (e.g., a left-hand response was always required for a left-sided target).

## Supplementary Information

Below is the link to the electronic supplementary material.

Supplementary Material 1

## Data Availability

The data is available at: https://osf.io/3sqyp/
