# Supplementary material for: ERP evidence of attentional selection outside of effective oculomotor range
Source: Exp Brain Res. 2025 Dec 23;244(1):16. doi: 10.1007/s00221-025-07219-0 (PMC12727773; doi:10.1007/s00221-025-07219-0)
Supplement: Supplementary file 1 — Supplementary Material 1 [file 221_2025_7219_MOESM1_ESM.docx]

**Supplement 1**

**Figure 1. Topography plots of lateralized ERPs.**


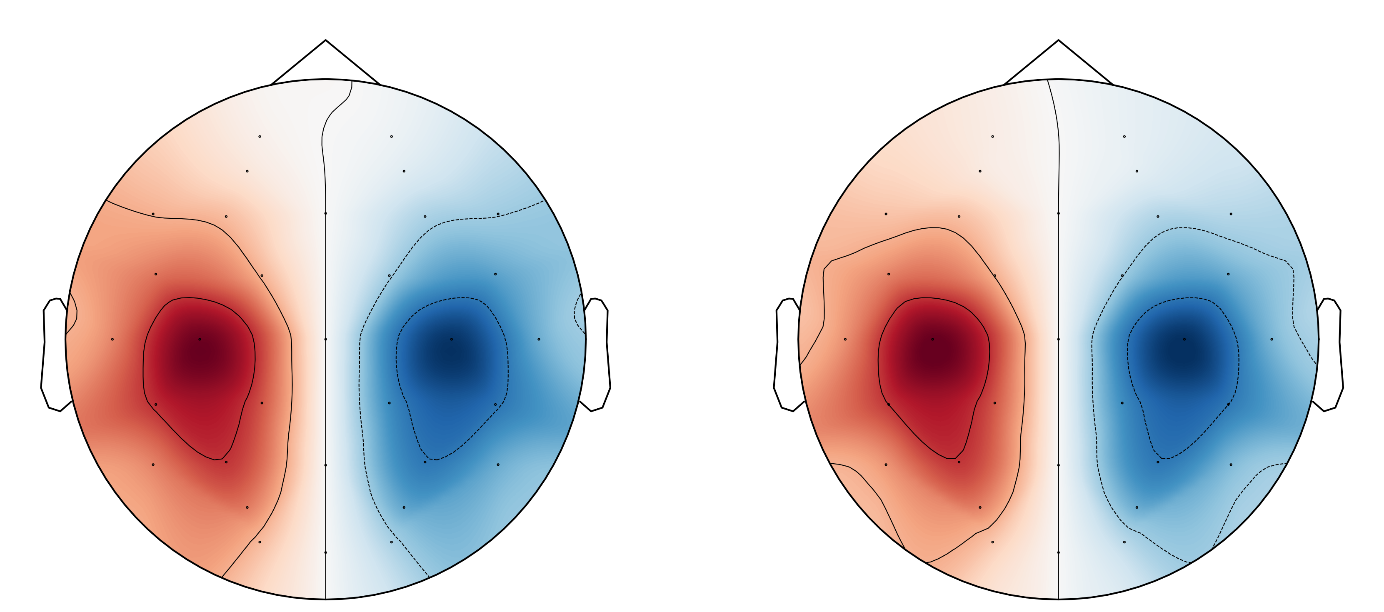


*Note.* Topographical plots from the frontal (right) and abducted (left) conditions in the 200–300 ms window are shown. Although a clear N2pc is visible at electrodes PO3 and PO4, the overall scalp distribution does not correspond to the typical N2pc topography. The maps appear to be dominated by the N2cc component, which may overshadow the concurrently present N2pc. Alternatively, the unexpected topographical shift may reflect somatosensory or visuomotor integration processes, as participants prepared a specific motor response for each target, eliminating the need for response selection once the target was identified.
